# EGCG, GCG, TFDG, or TSA Inhibiting Melanin Synthesis by Downregulating MC1R Expression

**DOI:** 10.3390/ijms241311017

**Published:** 2023-07-03

**Authors:** Wei Wang, Taimei Di, Weiwei Wang, Heyuan Jiang

**Affiliations:** 1 Key Laboratory of Biology, Genetics and Breeding of Special Economic Animals and Plants, Ministry of Agriculture and Rural Affairs, Tea Research Institute, Chinese Academy of Agricultural Sciences, 9 Meiling South Road, Xihu District, Hangzhou 310008, China; ww1040491839@163.com (W.W.);; 2 College of Horticulture, Fujian Agriculture and Forestry University, Cangshan District, Fuzhou 350002, China

**Keywords:** melanin, catechins and their dimers, α-MSH, MC1R, TYR

## Abstract

Without affecting cell viability, epigallocatechin gallate (EGCG), gallocatechin gallate (GCG), theaflavine-3,3′-digallate (TFDG), or theasinensin A (TSA) have been found to effectively reduce intracellular melanin content and tyrosinase (TYR) activity. However, studies on the anti-melanogenic mechanism of the above samples remain weak, and the activities of these samples in regulating melanogenesis at the molecular level lack comparison. Using B16F10 cells with the α-melanocyte-stimulating hormone (α-MSH) stimulation and without the α-MSH stimulation as models, the effects of EGCG, GCG, TFDG, or TSA on cell phenotypes and expression of key targets related to melanogenesis were studied. The results showed that α-MSH always promoted melanogenesis with or without adding the four samples. Meanwhile, the anti-melanogenic activities of the four samples were not affected by whether the α-MSH was added in the medium or not and the added time of the α-MSH. On this basis, the 100 µg/mL EGCG, GCG, TFDG, or TSA did not affect the TYR catalytic activity but inhibited melanin formation partly through downregulating the melanocortin 1 receptor (MC1R), microphthalmia-associated transcription factor (MITF), and the TYR family. The downregulation abilities of catechins on the TYR family and MITF expression were stronger than those of dimers at both the transcription and translation levels, while the ability of dimers to downregulate the MC1R expression was stronger than that of catechins at both the transcription and translation levels to some extent. The results of molecular docking showed that these four samples could stably bind to MC1R protein. Taken together, this study offered molecular mechanisms for the anti-melanogenic activity of the EGCG, GCG, TFDG, and TSA, as potential effective components against the UV-induced tanning reactions, and a key target (MC1R) was identified.

## 1. Introduction

Melanin, synthesized by the melanosomes in melanocytes [1], is widely found in animal hair and skin [2,3]. Melanin is a self-protection mechanism triggered by the body in response to external adverse stimuli, for example, ultraviolet radiation. A moderate amount of melanin is needed by the body, but the abnormal accumulation of melanin in the basal layer of the epidermis can cause uneven complexion, dark skin color, and various skin spots [4]. Meanwhile, the process of melanogenesis represents a potential cellular hazard [5]. Melanogenesis is generally overexpressed in malignant melanoma [6], which is malignantly transformed by melanocytes [7]. More and more whitening skin care products springing up on the market, as well as the booming medical beauty industry such as lightening skin spots by laser, reflect the urgent need of consumers to curb the abnormal accumulation of melanin. 

Solar ultraviolet radiation is a key external physical factor that impacts the biological effect of the skin. In a normal tanning reaction, ultraviolet radiation induces keratinocytes to produce an α-melanocyte-stimulating hormone (α-MSH), which then binds to the melanocortin 1 receptor (MC1R) to launch the signaling pathway dependent on cyclic adenosine monophosphate (cAMP). MC1R is expressed on normal and malignant melanocytes and is known as the α-MSH receptor due to its high affinity for the α-MSH [8,9]. MC1R is the most upstream Gs-protein-coupled receptor in the cAMP-dependent signaling pathway. Therefore, location defines the important effect of the MC1R in regulating melanogenesis. Microphthalmia-associated transcription factor (MITF) is one of the key transcription factors that regulate melanin synthesis and survival and proliferation of melanocytes [10,11,12]. Although melanin synthesis is regulated by multiple intracellular signaling pathways, all three of the most common pathways (cAMP-dependent signaling pathway, Wnt/β-catenin signaling pathway, and MAPK signaling pathway) share the final target on MITF, so MITF plays a crucial role in regulating melanogenesis. MITF will later activate the expression of protease related to melanin formation. Three enzymes in the tyrosinase (TYR) family—TYR, tyrosinase-related protein 1 (TRP1), and tyrosinase-related protein 2 (TRP2)—engage in the synthesis of melanin. Among them, TYR is a copper-containing metal oxidoreductase and is involved in the synthesis of two kinds of melanin (eumelanin and pheomelanin). TRP1 and TRP2 take part in the synthesis of eumelanin. TRP1 oxidizes 5, 6-dihydroxyindole-2-carboxylic acid (DHICA) and catalyzes the oxidative polymerization of DHICA to eumelanin. In addition, TRP1 directly participates in the formation of the ultrastructure of melanosomes and influences the growth, proliferation, and apoptosis of melanocytes [13]. TRP2 can quickly convert dopachrome into DHICA [14]. 

Tea is a healthy drink, which has become people’s consensus. Many reports have shown that the effective compounds of tea can inhibit the synthesis of melanin in melanocytes [15,16,17], inhibit the carcinogenesis of the skin induced by UVB [18,19,20,21,22], and provide photoprotection for skin [23,24]. Using the natural, green, plant-derived effective compounds extracted from tea as alternative ingredients for the anti-ultraviolet-induced tanning reactions has significant potential. Four compounds with the strongest anti-melanogenic activity, namely epigallocatechin gallate (EGCG), gallocatechin gallate (GCG), theaflavin-3,3′-gallate (TFDG), and theasinensin A (TSA), have been screened out from catechins and their dimeric oxidation products in our previous research [25]. The anti-melanogenic activity of these four compounds at 100 µg/mL was significantly stronger than or equal to that of kojic acid, a commonly positive control against melanogenesis [25]. The EGCG is the core compound of tea polyphenols with physiological activity and broad applications [26]. At the same concentration, the anti-melanogenic activity of GCG was stronger than or equal to that of the EGCG [25]. As ample dimers in black tea, the TFDG and TSA have been paid more and more attention by researchers, because they have abundant active groups and show good biological activity [27,28]. Understanding the molecular mechanisms that these four compounds exert in their anti-melanogenic activity could facilitate their application. Some studies have manifested that these four compounds inhibit melanin synthesis by acting on some key targets in the cAMP-dependent signaling pathway. A paper reported that catechins regulate melanogenesis in B16F10 cells through the cAMP/CREB/MITF pathway and ultimately significantly inhibit the intracellular TYR activity and reduce melanin content, with the activity ranking from strong to weak as epicatechin gallate (ECG) > EGCG > GCG > β-arbutin [29]. The 10 μM TFDG also decreased the melanin content in the α-MSH-induced cells by downregulating the expression of TYR mRNA and protein, and its activity was stronger than that of the EGCG, epigallocatechin, ECG, epicatechin, and kojic acid at the same molarity [30]. TSA was reported to downregulate the mRNA expression of *TYR*, *TRP1,* and *TRP2*, suppress the activities of certain proteins (CREB, protein kinase A, TYR, and MITF), and reduce the quantities of cAMP in the α-MSH-induced B16F10 cells [17]. However, the above studies about anti-melanogenic molecular mechanisms rarely involve MC1R. Meanwhile, there is a lack of comparison between catechins and dimers at the molecular level. In the literature, the α-MSH was frequently used to stimulate cells. On the one hand, the α-MSH can increase the TYR activity and the melanin content in the cells themselves, which makes it easy to amplify the differences among various treatments to heighten the comparability of the data. On the other hand, the α-MSH represents the effects of ultraviolet on cells to some extent. Notably, whether the α-MSH and the sample, the two components added exogenously, interact with each other has not been reported. Finally, whether the activity of the sample to hinder melanin synthesis is caused by directly inhibiting the catalytic activity of enzymes or by disturbing the synthesis of enzymes related to melanogenesis is also an interesting question worth exploring.

Based on the above problems to be solved, B16F10 cells were cultured by the samples (100 µg/mL EGCG, GCG, TFDG, and TSA) for 2 h, and then the α-MSH was used to stimulate the cells or not in the presence of the samples to build a cell model stimulated by the α-MSH and a cell model not stimulated by the α-MSH, respectively. Whether the α-MSH and the sample interfere with each other was first investigated. Then, the influence of the four samples at the same concentration on the TYR catalytic activity and intracellular TYR activity was analyzed. Next, the effects of the four samples on the expression of MC1R were studied, and the ability of the samples to regulate the key targets related to melanogenesis was compared. Finally, molecular docking was used to verify the biding of the four samples to the MC1R protein. The above studies could provide a new basis for the effectiveness and practicability of the α-MSH-induced cell model in research related to the anti-melanogenic activity of the compounds, offer molecular mechanisms for the anti-melanogenic activity of the EGCG, GCG, TFDG, and TSA, and supply a new target to treat hyperpigmentation.

## 2. Results

### 2.1. Samples Did Not Deactivate α-MSH but Inhibited the Intracellular Melanin Production Induced by α-MSH

**Figure 1:**
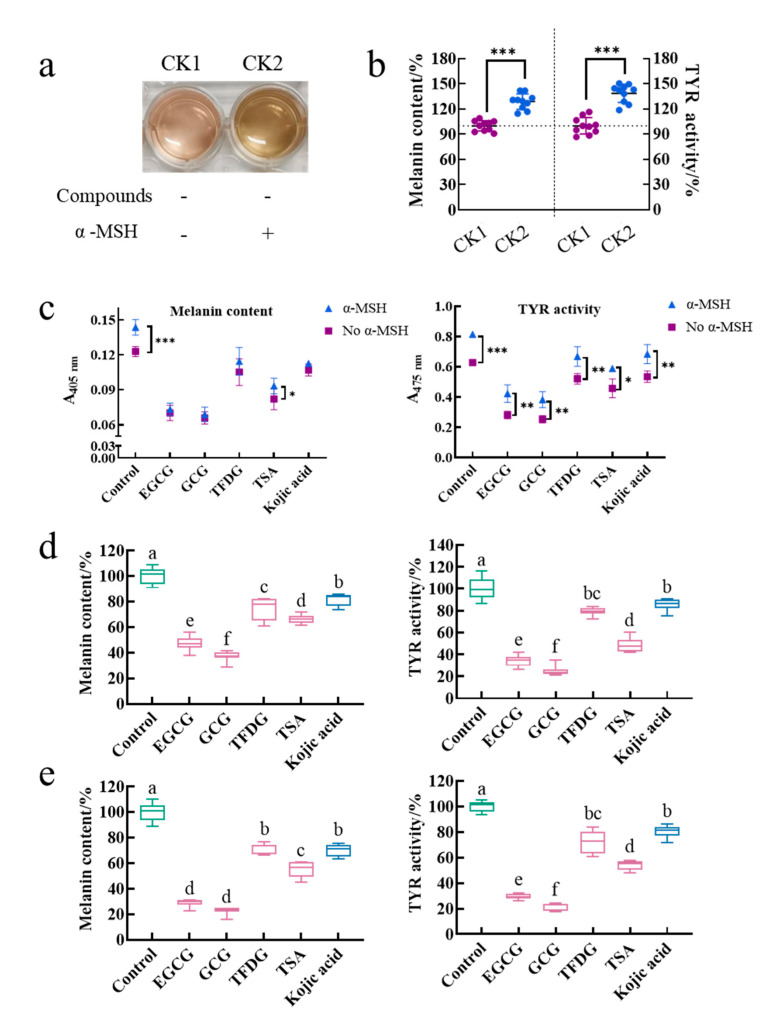
Epigallocatechin gallate (EGCG), gallocatechin gallate (GCG), theaflavine-3,3’-digallate (TFDG), and theasinensin A (TSA) did not deactivate α-melanocyte-stimulating hormone (α-MSH) but inhibited the production of melanin induced by α-MSH in B16F10 cells. B16F10 cells were pretreated with EGCG, GCG, TFDG, or TSA for 2 h. Then they were further cultured for 48 h (cells without α-MSH stimulation) or further stimulated with α-MSH for 48 h in the presence of EGCG, GCG, TFDG, or TSA (cells with α-MSH stimulation). (**a**,**b**) The α-MSH stimulation of cells resulted in the darkening of the medium, an increase in intracellular melanin content, and a boost in intracellular tyrosinase (TYR) activity. The treatment group without α-MSH and a sample was referred to as control 1 (CK1), and the treatment group with α-MSH but without a sample was referred to as control 2 (CK2). (**c**) α-MSH increased the absorbance of A_405_ in the melanin content assay and A_475_ in the enzymatic reaction assay. (**d**) EGCG, GCG, TFDG, and TSA significantly reduced the intracellular melanin content and intracellular TYR activity of cells without α-MSH stimulation. (**e**) EGCG, GCG, TFDG, and TSA significantly reduced the intracellular melanin content and intracellular TYR activity of cells with α-MSH stimulation. Data are mean ± SD; * *p* < 0.05, ** *p* < 0.01, and *** *p* < 0.001; ^a,b,c,d,e,f^ different letters above the boxplot indicate significant differences (*p* < 0.05).

The treatment group without the α-MSH and a sample was referred to as control 1 (CK1), and the treatment group with the α-MSH but without a sample was referred to as control 2 (CK2). The effect of the α-MSH on the physiological activity of cells was investigated by comparing the color of the medium, intracellular melanin content, and intracellular TYR activity between CK1 and CK2. The medium color of CK2 was darker than that of CK1 (Figure 1a). Meanwhile, CK2 had significantly higher intracellular melanin content (129 ± 9%) and intracellular TYR activity (135 ± 15%) than those of CK1 (100 ± 6% and 100 ± 10%, respectively, *p* < 0.001, Figure 1b). The cell viability of CK1 (96 ± 5%) was the same as that of CK2 (100%, Table S1). That is, the α-MSH did not affect cell viability. Results suggested that the α-MSH increased the intracellular melanin content by promoting the intracellular TYR activity.

Is the ability of the α-MSH to promote melanin synthesis interfered by the samples? In this study, after being cultured by these four samples for 2 h, the cells were stimulated by the α-MSH in the presence of the samples for another 48 h. During this process, because the sample acted on the cells before the α-MSH and treated the cells together with the α-MSH for another 48 h, whether the sample inactivating the α-MSH or whether the sample inhibiting the α-MSH binding to the MC1R and thus blocking the initiation of the cAMP-dependent signaling pathway could be investigated by comparing the absorbance of the melanin content assay and the TYR activity assay between the treatment induced by the α-MSH and the treatment not induced by the α-MSH under the same sample treatment. As shown in Figure 1c, the α-MSH significantly increased the absorbance of 405 nm in the control and the TSA group, while the absorbance of 475 nm in each treatment group was significantly enhanced by the α-MSH. That is to say, the α-MSH could increase the melanin content and TYR activity in the sample treatment group. Therefore, the α-MSH always promoted melanin synthesis with or without the addition of these four samples. In other words, firstly, the α-MSH is not inactivated by these four samples. Secondly, the α-MSH successfully binds to MC1R. Finally, the cAMP-dependent signaling pathway is activated.

The purpose of adding the α-MSH to the medium was to construct a cell model with strong melanin-producing capacity, thereby amplifying the physiological activity of the cells and facilitating the functional comparison of different samples. Is the activity of these four samples disturbed by the α-MSH during the modeling process? To investigate this issue, two cell models (without α-MSH-stimulation and with α-MSH-stimulation) were introduced in this experiment. These two cell models were cultured by these four samples under the same conditions for a while. Then, the effects of a sample on each model were observed and compared. The results showed that EGCG, GCG, TFDG, and TSA not only significantly reduced the intracellular melanin content and inhibited the intracellular TYR activity in the B16F10 cells without the α-MSH stimulation (Figure 1d, *p* < 0.05) but also played the same role in the B16F10 cells stimulated by the α-MSH (Figure 1e, *p* < 0.05). At the same time, the ability of these four samples to reduce melanin content and TYR activity was always GCG ≥ EGCG > TSA > TFDG ≥ kojic acid (*p* < 0.05), which was consistent with the results in our previous study (α-MSH- and compound-treated cells at the same time) [25]. Therefore, the anti-melanogenic activity of these four samples was not affected by whether the α-MSH was added in the medium or not and the time of the α-MSH addition. The premise of the above results was that the 100 µg/mL EGCG, GCG, TFDG, and TSA with the anti-melanogenic activity did not interfere with the α-MSH-stimulated cell viability (cell viability exceeded 90% in all groups, Table S1).

### 2.2. Samples Did Not Inhibit the Catalytic Activity of TYR

As shown in Figure 1e, the EGCG, GCG, TFDG, and TSA significantly inhibited the α-MSH-stimulated intracellular melanin content and intracellular TYR activity as kojic acid does. EGCG (29 ± 3% melanin content, 29 ± 2% TYR activity), GCG (23 ± 3% melanin content, 20 ± 3% TYR activity), and TSA (55 ± 6% melanin content, 54 ± 3%TYR activity) were more effective compared with kojic acid (70 ± 5% melanin content, 80 ± 5% TYR activity, *p* < 0.05), while TFDG (70 ± 5% melanin content, 72 ± 9% TYR activity) had no significant difference from kojic acid. Is the effect of these four samples related to the direct inhibition of the TYR catalytic activity in the B16F10 cells?

**Figure 2:**
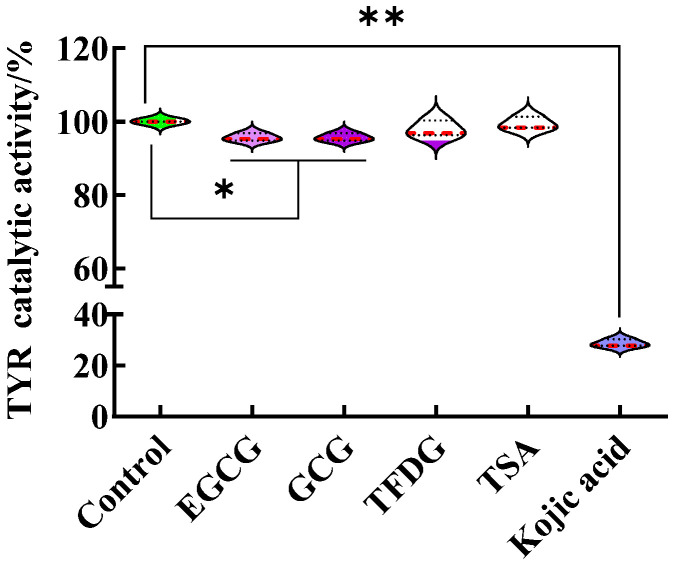
Influence of 100 µg/mL EGCG, GCG, TFDG, and TSA on the TYR catalytic activity. The treatment group without samples was used as a control (TYR catalytic activity was 100%). In the non-cellular system, the effect of samples on the activity of 3,4-dihydroxyphenylalanine (L-DOPA) oxidation by TYR was observed; * *p* < 0.05, ** *p* < 0.01.

In this part, the B16F10 cells at the exponential phase were collected, cracked, and centrifuged. The obtained lysate supernatant was used as a crude TYR solution, and the influence of the sample on the ability of the crude enzyme to oxidize 3,4-dihydroxyphenylalanine (L-DOPA) was studied in the non-cellular system. Taking the treatment without samples as the control (TYR catalytic activity was 100%), although statistically significant differences were found in the TYR catalytic activity between the EGCG or GCG and the control, the catalytic activities of TYR treated by the EGCG, GCG, TFDG, and TSA were above 95% (Figure 2, Table S2). That is, these four samples at 100 µg/mL did not affect the catalytic activity of TYR. The results indicated that the significant decrease in the intracellular TYR activity induced by the samples (100 µg/mL EGCG, GCG, TFDG, and TSA) as shown in Figure 1e was not caused by the direct inhibition of the TYR catalytic activity, but the samples indirectly inhibited the activity of intracellular TYR by affecting a complex intracellular signaling pathway, such as the synthesis of new TYR. Unlike catechins and their dimers, 100 µg/mL kojic acid could directly inhibit the TYR catalytic activity (29 ± 1% TYR catalytic activity, *p* < 0.01), which might be one of the mechanisms by which kojic acid reduced the intracellular TYR activity and melanin content in the B16F10 cells.

### 2.3. Effects of Samples on Expression of TYR, TRP1, and TRP2 at Transcription and Translation Levels

Melanogenesis mainly involves three enzymes in the TYR family, namely TYR, TRP1, and TRP2, which are membrane-bound glycoproteins [31]. TYR has dual activities of tyrosine hydroxylase and dopa oxidase, catalyzing key rate-limiting steps in the biosynthesis pathway of eumelanin and pheomelanin [32,33]. TRP1 and TRP2 are required in the production of eumelanin but not pheomelanin [33]. In addition, TRP1 and TRP2 are involved in stabilizing TYR, regulating the catalytic activity of TYR, and maintaining the melanosome structure [34].

**Figure 3:**
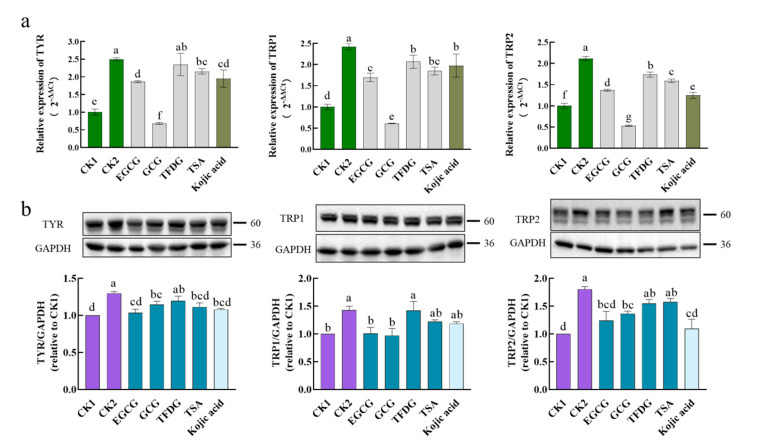
Effect of EGCG, GCG, TFDG, or TSA on expression of TYR, tyrosinase-related protein 1 (TRP1), and tyrosinase-related protein 2 (TRP2) mRNA and protein. B16F10 cells were pretreated with EGCG, GCG, TFDG, or TSA for 2 h and stimulated with α-MSH for 24 h in the presence of EGCG, GCG, TFDG, or TSA. The treatment group without α-MSH and a sample was referred to as CK1, and the treatment group with α-MSH but without a sample was referred to as CK2. (**a**) Cell lysates were prepared with an EASY spin Plus cell RNA rapid extraction kit. Total RNAs were subjected to qPCR analysis of *TYR*, *TRP1,* or *TRP2* with *β-actin* as an internal control. Data are mean ± SD. (**b**) Protein extracts were prepared by lysing cells with radioimmunoprecipitation assay (RIPA) lysate containing protease inhibitors and phosphatase inhibitors. Total proteins were subjected to Western blot analysis of TYR, TRP1, or TRP2 with GAPDH as an internal control. Data are mean ± SEM; ^a,b,c,d,e,f,g^ different letters above the column indicate significant differences (*p* < 0.05).

To discover the anti-melanogenic mechanism of these four samples, the genes and proteins expression levels of TYR, TRP1, and TRP2 were detected first. As shown in Figure 3, the α-MSH significantly upregulated the expression of the TYR, TRP1, and TRP2 genes and proteins, indicating that the α-MSH enhanced the intracellular TYR activity and melanin content at the level of transcription and translation. This further validated the results in Figure 1b.

Compared with CK2, kojic acid could significantly inhibit the mRNA expression of *TYR*, *TRP1*, and *TRP2*, as well as the protein expression of TYR and TRP2. Therefore, as a skin-lightening agent, kojic acid plays a whitening role at least partly by downregulating the mRNA and protein expression of the TYR family. This means that kojic acid is a suitable positive control. In terms of the four samples, the EGCG and GCG could significantly inhibit the mRNA and protein expression of the TYR family (TYR, TRP1, TRP2) with CK2 as a reference; the TFDG significantly inhibited the mRNAs expression of *TRP1* and *TRP2*, while the TSA significantly inhibited the mRNAs expression of *TYR, TRP1,* and *TRP2* and the protein expression of TYR. These results indicated that these four samples did hinder the synthesis of new enzymes associated with melanin production in cells, thus inhibiting the intracellular TYR activity and reducing the melanin content.

The ability of these four samples to downregulate mRNA or protein expression was then compared. When it came to downregulation of the mRNA expression levels, GCG was always significantly stronger than kojic acid; TFDG and TSA were less than or equal to kojic acid, and the ability of the EGCG was different from that of kojic acid in different mRNAs. Additionally, the ability of these four samples was always ranked as GCG > EGCG ≥ TSA ≥ TFDG in downregulating the expression of the *TYR, TRP1,* and *TRP2*, which was consistent with the phenotypic results (Figure 1e), and GCG reduced the expression levels of these three genes to lower than CK1. When it came to downregulation of the TYR protein expression level, only TFDG could not significantly inhibit the TYR expression. There was no significant difference among the EGCG, GCG, TSA, and kojic acid, and there was also no significant difference among the GCG, TFDG, TSA, and kojic acid. The EGCG showed the strongest activity, downregulating TYR to the CK1 level, which was significantly stronger than that of TFDG. Only the EGCG and GCG could significantly inhibit the TRP1 protein expression to the CK1 level, but there was no significant difference between the EGCG and GCG. In addition, only the EGCG, GCG, and kojic acid could significantly downregulate the expression of the TRP2 protein, but there was no significant difference among them.

Overall, the EGCG, GCG, TFDG, and TSA reduced the amount of the TYR family in the cell model by downregulating the expression of the α-MSH-activated TYR, TRP1, and TRP2 mRNA and protein. That is these four samples reduced the melanin synthesis by inhibiting the additional production of enzymes (TYR, TRP1, and TRP2) in the cells. Compared with the translation level, the differences in the transcription level among the four samples were more obvious, and an accordant trend was displayed. The downregulating ability of catechins was stronger than that of dimers at both the transcription and translation levels.

### 2.4. Effects of Samples on Expression of MITF at Transcription and Translation Levels

**Figure 4:**
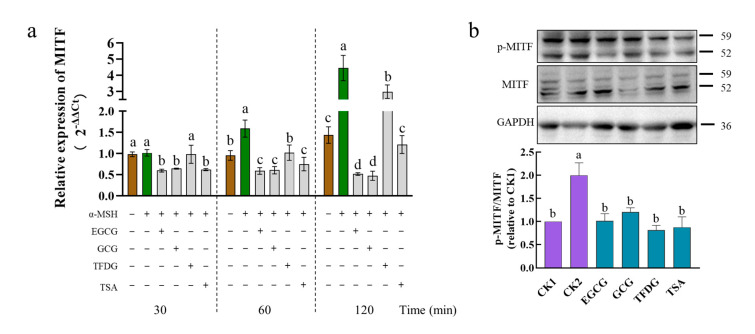
Effect of EGCG, GCG, TFDG, or TSA on expression of microphthalmia-associated transcription factor (MITF) mRNA and protein. B16F10 cells were pretreated with EGCG, GCG, TFDG, or TSA for 2 h and stimulated with α-MSH for 30–120 min (**a**) or 1 h (**b**) in the presence of EGCG, GCG, TFDG, or TSA. The implementation and data analysis of qPCR and Western blot were the same as those in Figure 3; ^a,b,c,d^ different letters above the column indicate significant differences (*p* < 0.05).

MITF is the most crucial transcriptional regulatory factor that regulates the expression of the *TYR*, *TRP1,* and *TRP2* genes. Upon exposure to the α-MSH alone, the B16F10 cells markedly increased the mRNA levels of *MITF* at 60 min to 120 min but not 30 min (Figure 4a). Treatment with EGCG, GCG, or TSA suppressed the mRNA levels of the *MITF* gene at 30 min to 120 min after exposure to the α-MSH, as did the TFDG at 60 min to 120 min but not 30 min. After being stimulated by the α-MSH for 1 h, the α-MSH significantly upregulated the protein expression and the phosphorylation level of MITF, while the four samples significantly downregulated the protein expression and phosphorylation level of the MITF induced by the α-MSH (Figure 4b and Figure S1). These effects lasted up to 4 h after the exposure to the α-MSH (Figure S2). The above results indicated that EGCG, GCG, TFDG, or TSA could downregulate the expression of the α-MSH-activated MITF genes and proteins, as well as the MITF phosphorylation, and thereby downregulate the expression of TYR, TRP1, and TRP2 at both the transcriptional and translational levels.

The activities of these four samples were further compared. The downregulation of the *MITF* expression in the sample groups was always EGCG = GCG ≥ TSA > TFDG from 30 to 120 min. With time extending, the difference among the treatment groups became more obvious (Figure 4a). 

After the cells were treated with the α-MSH and the samples together for 1 h, although there was no significant difference among the four samples, these samples all could downregulate MITF phosphorylation to the no-α-MSH-stimulation level (CK1, Figure 4b). The protein expression of MITF in the four samples also reached the CK1 level, the expression level of MITF in the EGCG group was the lowest among them and significantly lower than that in the TFDG. There was no significant difference among the EGCG, GCG, and TSA, and no significant difference among the GCG, TFDG, and TSA groups (Figure S1). The experiment went on for 4 h, no significant difference was found among the sample groups, and the MITF phosphorylation levels of the four samples were between those of CK1 and CK2 (Figure S2). The expression level of the MITF protein in the GCG treatment group was the lowest, which was significantly lower than that in CK1, but had no significant difference from that in the EGCG and TSA treatment groups. The EGCG, TFDG, TSA, and CK1 had no significant differences from each other.

In general, the effect of catechins on the downregulation of the MITF gene and protein expression was stronger than that of dimers, but there was no significant difference among the four samples in the MITF phosphorylation level. The difference in the transcription level among the treatment groups was more obvious than the translation level.

### 2.5. Effects of Samples on Expression of MC1R at Transcription and Translation Levels

MC1R is a seven-transmembrane G-protein-coupled receptor on melanocytes that is activated by the α-MSH [35]. Due to its high expression in malignant melanoma compared with normal organs and tissues, it is often used as a biomarker for melanoma, so it is considered a potential target for the selective delivery of melanoma therapeutics [36].

**Figure 5:**
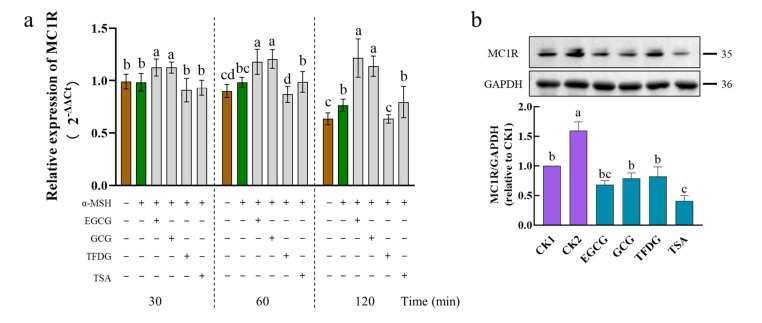
Effect of EGCG, GCG, TFDG, or TSA on expression of melanocortin 1 receptor (MC1R) mRNA and protein. B16F10 cells were pretreated with EGCG, GCG, TFDG, or TSA for 2 h and stimulated with α-MSH for 30–120 min (**a**) or 1 h (**b**) in the presence of EGCG, GCG, TFDG, or TSA. The implementation and data analysis of qPCR and Western blot were the same as those in Figure 3; ^a,b,c,d^ different letters above the column indicate significant differences (*p* < 0.05).

After the α-MSH stimulation, the B16F10 cells significantly upregulated the *MC1R* mRNA levels at 120 min. The mRNA level of *MC1R* was further significantly upregulated by the EGCG and GCG during 30–120 min. At 60–120 min, the TFDG significantly downregulated the expression of *MC1R*. At 30–120 min, the TSA had no significant effect on the *MC1R* expression (Figure 5a). Interestingly, the four samples showed three different effects on the *MC1R* expression.

At the translation level (Figure 5b), MC1R was expressed in a cell model (CK1) that was not stimulated by the α-MSH, possibly because of the presence of the endogenous α-MSH [37,38]. The protein expression of MC1R was significantly upregulated by the α-MSH stimulation (CK2) but significantly downregulated by the four samples. The downregulated of the MC1R protein by the samples could be continued for 4 h (Figure S2). 

After the cells were treated by the α-MSH and a sample together for 1 h, the TSA had the strongest ability to downregulate the MC1R, which was significantly stronger than that of the GCG, TFDG, and CK1 but had no significant difference from that of the EGCG. The remaining three samples showed no significant difference between each other and CK1 (Figure 5b), indicating that these samples had a strong ability to downregulate the expression of MC1R to the level of no α-MSH stimulation. These samples maintained this ability for 4 h and still had no significant difference from CK1 (EGCG, GCG, and TSA), or it was significantly stronger than that of CK1 (TFDG). At this point, the TFDG group had the strongest ability to downregulate the MC1R expression, and the remaining three samples had no significant difference from each other. The ability of dimers to downregulate the MC1R expression was stronger than that of the catechins.

The four samples had different effects on the expression of MC1R. Although the EGCG and GCG upregulated the *MC1R* expression at the transcriptional level (30–120 min), they significantly downregulated the MC1R expression at the translational level (1 h and 4 h). Compared with the transcription level, the translation level is more representative of phenotypic changes, which may be the reason why EGCG and GCG significantly inhibit the intracellular TYR activity and reduce melanin content. TFDG could significantly downregulate the MC1R expression at both the transcription and translation levels, while the TSA could downregulate the MC1R at the translation level. The reasons for the differences among the four samples need to be further explored.

### 2.6. Docking Results

Molecular docking was used to certify the binding of these four samples to the MC1R protein. In molecular docking, the XP mode is flexible docking (both the protein and the ligand are flexible) and is the most refined computational mode, which can be used to perform higher-resolution molecular docking calculations on specific targets. The MM-GBSA analysis results could further verify the results of the XP docking. The XP docking results and the MM-GBSA analysis results were referred to XP Gscore and MM-GBSA dG Bind, respectively. The XP Gscore indicates the docking score, which can reflect the binding free energy. The MM-GBSA dG Bind represents the binding free energy, which is an absolute quantification of the binding strength of the ligand and protein. When the XP Gscore is less than −6, it is generally believed to have stable binding between the ligand and protein. When the value of MM-GBSA dG Bind is less than −30 kcal/mol, it indicates that the binding free energy is low, and the binding between the ligand and protein is stable.

A comprehensive analysis of the results of the XP docking and MM-GBSA analysis showed that the TFDG and MC1R protein had the best docking performance, with an XP Gscore of −10.121 and MM-GBSA dG Bind of −35.1 kcal/mol (Table 1). The second was the EGCG, whose XP Gscore was −7.844, and MM-GBSA dG Bind was −36.3 kcal/mol. Both the docking score and free binding energy were low, indicating that the docking between the EGCG and MC1R protein was stable enough. In addition, the MM-GBSA dG Bind values of the GCG and TSA were more than −30 kcal/mol, but their XP Gscore was less than −6 for both (we gave priority to the XP Gscore). Therefore, the binding of these two compounds to the MC1R protein was relatively stable. The stable binding ability of the TFDG, EGCG, GCG, and TSA to the MC1R protein was consistent with the ability of these samples to downregulate the MC1R expression in Figure S2b. To sum up, all four samples could stably bind to the MC1R protein, which proved that these samples exerted the anti-melanogenic activity by regulating the expression of the MC1R protein to a certain extent.

**Figure 6:**
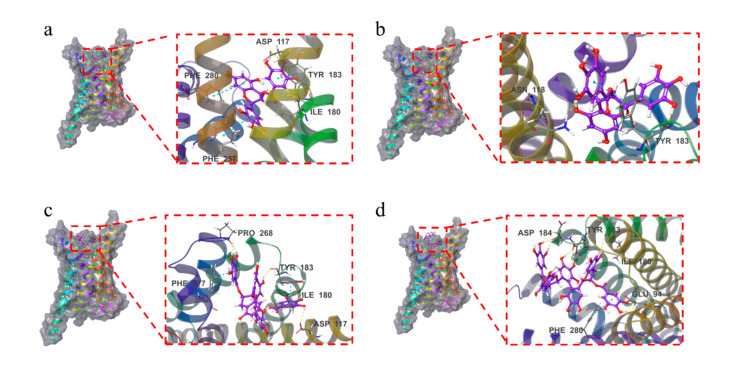
Three-dimensional images of the four compounds docking with MC1R protein: (**a**) EGCG, (**b**) GCG, (**c**) TFDG, and (**d**) TSA. Hydrogen bonds are represented by yellow dotted line and π-π bonds are represented by blue dotted line.

As shown in Figure 6and Figure S3, the four compounds were all bound to the surface of the active pocket of the MC1R protein.

The EGCG formed hydrophobic forces with the MC1R protein residues PHE257, LEU284, MET128, CYS125, and TYR183. Additionally, the EGCG formed a hydrogen bond with residues ILE180 and ASP117 and a π-π bond with residues TYR183, PHE280, and PHE257.

The GCG formed hydrophobic forces with the MC1R protein residues CYS125, MET128, PHE257, LEU192, LEU261, etc. The GCG formed a hydrogen bond with the MC1R protein residue ASN118 while forming a hydrogen bond and a π-π bond with TYR183.

The TFDG formed hydrophobic forces with the MC1R protein residues PHE277, PRO268, CYS267, ILE264, LEU189, etc. The TFDG formed a hydrogen bond with residue ASP117, two hydrogen bonds with residues PRO268 and ILE180 and a π-π bond with residue PHE277. The TFDG also formed a hydrogen bond and a π-π bond with residue TYR183.

The TSA formed a hydrophobic force with residue VAL186, a hydrogen bond with residue ILE180, and two hydrogen bonds with residues ASP184 and GLU94. The TSA formed a π-π bond with residue PHE280 and two hydrogen bonds and one π-π bond with residue TYR183.

## 3. Discussion

Abnormal accumulation of melanin can grievously affect the appearance of people, and upregulated melanogenesis is one of the characteristics of melanoma [5,39]. Utilizing natural plant components is an important means to inhibit the abnormal synthesis of melanin. Catechins and their dimers have been discovered as components that showed effective anti-melanogenic activity in recent years. However, some problems about the anti-melanogenic activity of catechins and their dimers are waiting to be solved. For example, is there any interaction between the α-MSH and the sample? What is the relationship between the anti-melanogenic activity of the sample and the inhibition of the TYR catalytic activity or intracellular TYR activity? Compared with the known signaling pathway of melanin formation, the molecular mechanism of inhibiting melanin synthesis by catechins and their dimers is still relatively weak. Additionally, comparisons of activity (molecular level) between the samples, especially between catechins and dimers, are relatively rare. In this context, this paper explored these questions step by step. The mechanism of the EGCG, GCG, TFDG, and TSA regulating melanogenesis was enriched, and a new target for treating hyperpigmentation was provided.

The α-MSH-induced cell model used in this study involves the important signaling pathway of melanin formation, namely the cAMP-dependent signaling pathway. It is also known as the MC1R/α-MSH signaling pathway. After binding with MC1R, the α-MSH activates the intracellular cAMP-dependent signaling pathway and increases the expression of *TYR*, *TRP1,* and *TRP2* through the upregulation of *MITF*, thus promoting intracellular melanin synthesis. The melanogenic effect of the α-MSH has been demonstrated several times [17,38], and the consistent results obtained in our experiment (darker medium color, more melanin content, and higher intracellular TYR activity in CK2 compared with CK1) indicated that the modeling was successful. 

It has become a popular choice for researchers to amplify the melanogenic activity of cells by adding the α-MSH to the medium to easily compare the differences of indicators under different sample treatments. Whether adding the α-MSH affects the anti-melanogenic activity of the sample is an interesting question. In addition, different works in the literature have slightly different α-MSH addition times when establishing cell models stimulated by the α-MSH. In some methods, cells were simultaneously treated by the α-MSH and the samples [17]. In other methods, the α-MSH was added after cells were cultured by a sample for 2 h [33]. The former is likely to be less time-consuming and easier to be operated. The latter considers the possibility of interaction between the α-MSH and the sample. So, will the addition time of the α-MSH affect the anti-melanogenic activity of the samples and consequently the comparability of different experimental results? Without interfering with cell viability, this study found that the 100 µg/mL EGCG, GCG, TFDG, and TSA significantly inhibited melanin production in the B16F10 cells, regardless of whether the α-MSH was added in the medium, or when the α-MSH was added. 

Interestingly, the 100 µg/mL samples (EGCG, GCG, TFDG, and TSA) did not affect the TYR catalytic activity. In addition, it was found that the α-MSH could always promote melanin synthesis with or without these four samples added, which means that adding these four samples did not deactivate the α-MSH or block the binding of the α-MSH to MC1R. These results suggest that the decrease in melanin content and TYR activity in the cells cultured by a complete culture medium containing the samples may be due to the regulation of the signaling pathways by these four samples. Here, we discuss a mechanism for treating hyperpigmentation disorders by EGCG, GCG, TFDG, and TSA through regulating the most upstream Gs-protein-coupled receptor (MC1R) in the cAMP-dependent signaling pathway.

The MC1R is known as the α-MSH receptor. The α-MSH binding to the MC1R will initiate melanin synthesis. In the absence of MC1R, the α-MSH cannot bind to the MC1R and therefore cannot promote melanin production, despite the UV-induced production of the α-MSH [40]. The pinna of the mouse ear is similar to human skin, and the epidermis has melanocytes. D‘Orazio et al. [40] compared the UV-tanning response of wild-type C57BL/6 mice possessing intact MSH pathway (*Mc1r*^E/E^) with the MSH receptor inactivated in a mutant mice (*Mc1r*^e/e^). It was found that the UV-induced hyperpigmentation was observed both visually and microscopically in the ears of the *Mc1r*^E/E^ mice. However, in the ears of the *Mc1r*^e/e^ mice, pigmentation was not affected by UV irradiation, and there was no significant difference among the treatment groups. Interestingly, the *Mc1r*^E/E^ and *Mc1r*^e/e^ had the same number of melanocytes. These results confirm the importance of the MC1R functioning in the α-MSH-induced hyperpigmentation. Lerner and McGuire conducted research showing that injecting melanocortin into human subjects enhanced skin darkening [41]. An injection of a powerful α-MSH analogue, NDP-MSH, into the body without sunlight can also cause increased pigmentation [42]. In addition, the expression of the *TYR* and *MC1R* genes was increased in differentiated melanocytes after exposure to narrow-band ultraviolet B (NB-UVB) radiation [43]. Treatment of melanocytes with 10^−7^ M α-MSH also resulted in the increased expression of *MC1R* mRNA [44], which means that the receptor expression will be upregulated after binding of the ligand to the receptor. These results indicated that both ultraviolet irradiation and α-MSH induction could lead to the upregulation of the *MC1R* expression and promote hyperpigmentation. After MC1R knockout, the mRNA expressions of *MC1R* and *TYR* were significantly downregulated [43]. Our results are consistent with the above results. The α-MSH stimulation significantly increased the expression levels of the MC1R gene and protein, which was consistent with the phenotype of the enhanced TYR activity and increased melanin content after the α-MSH induction. These results indicate that the α-MSH can promote the TYR activity and melanin synthesis by heightening the MC1R expression. At the transcription level, the four samples showed three different effects on the *MC1R* expression. However, at the translation level, the EGCG, GCG, TFDG, and TSA all significantly downregulated the α-MSH-activated MC1R protein expression. Specifically, the EGCG, GCG, TFDG, and TSA downregulated the MC1R protein expression to no-α-MSH-stimulation levels or even lower than that of the no-α-MSH-stimulation group (CK1) from 1 h to 4 h after the α-MSH stimulation, which indicates that these four samples could effectively reverse the upward expression of MC1R induced by the α-MSH. The results of the XP docking and MM-GBSA analysis showed that these four samples could stably bind to the MC1R protein. These results of our study pushed the target of the EGCG, GCG, TFDG, and TSA regulating the cAMP-dependent signaling pathway to MC1R. Certainly, some interesting results, the protein expression results of the EGCG and GCG being consistent with their phenotypes, but their mRNA expression results being opposite to their phenotypes, need to be further explored. Fang et al. [38] showed that in the normal skin melanocytes (PIG1 cells) with the α-MSH-induced high expression of melanin, niacinamide and tea polyphenol complex significantly inhibited the α-MSH-induced the upregulation of the MC1R expression. This further confirms the reliability of our results. Chen [45] suggested that tea polyphenols join in regulating the MC1R/α-MSH signaling pathway by directly inhibiting the α-MSH rather than downregulating the MC1R protein level. The reason for the contradiction between their result and ours may be that tea polyphenols are complex, rather than a single component, and their effects on the cell signaling pathways are more complex. We also compared the expression levels of MC1R in different sample treatments. From 1 h to 4 h after the α-MSH stimulation, the ability of dimers to downregulate the MC1R expression was stronger than that of catechins at both the transcription and translation levels to some extent, which was not consistent with the phenotype results (Figure 1e). These results implied that there must be other pathways involved in regulating the expression of the downstream MITF and TYR families, which further influence the intracellular TYR activity and melanin content.

MITF is an important transcription factor regulating the *TYR* family. After entering the nucleus, MITF can be phosphorylated and later bind to M-BOX and B-BOX in the promoter region of the *TYR* family, thus upregulating the transcription of the *TYR* family [46,47]. Therefore, the upregulation of MITF can activate the expression of melanin-related enzymes, thus promoting the generation of melanin. Studies have shown that treating ultraviolet-radiated melanocytes with the α-MSH increases the phosphorylated and nonphosphorylated MITF levels [48]. In one report, EGCG, ECG, and GCG downregulated the expression of the MITF gene and protein in B16F10 cells, with significant differences compared with the control group (*p* < 0.01) [49]. Kim E et al. [16] reported that besides inhibiting the TYR activity, the generation of MITF was also significantly reduced after the EGCG treatment of the Mel-Ab melanocytes. Chen [45] showed that UVA irradiation significantly increased the protein expression level of MITF, while tea polyphenols (5–15 μg/mL) significantly inhibited the UVA-induced increase in the protein expression level of MITF. In unirradiated cells, tea polyphenols (5–15 μg/mL) could also significantly inhibit the protein expression of MITF [17]. The results of our study are consistent with the above reports. That is, the α-MSH upregulated the expression of MITF at both the transcription and translation levels and promoted the phosphorylation of MITF. The EGCG, GCG, TFDG, or TSA could downregulate the expression of the α-MSH-activated MITF gene and protein and downregulate the phosphorylation level of MITF. In addition, the effect of catechins on downregulating the MITF gene and protein expression was stronger than that of dimers, which was consistent with the phenotype results (Figure 1e). However, there was no significant difference among the four samples in the MITF phosphorylation level. Therefore, it can be speculated that the ability of a sample to inhibit the intracellular TYR activity and reduce melanin content can be predicted by the expression levels of the MITF protein and gene to a certain degree.

The TYR, TRP1, and TRP2 are located in the same multienzyme complex on the membrane of melanosome and interact with each other to jointly regulate melanogenesis in melanocytes [38]. Studies have shown that adding the α-MSH upregulates the protein and mRNA expression levels of TYR, TRP1, and TRP2, while adding these four samples downregulates the protein and mRNA expression levels of these enzymes [34]. The EGCG, ECG, and GCG could significantly downregulate the expression of the TYR, TRP1, and TRP2 genes and proteins (*p* < 0.01) [49]. Tea polyphenols (5–15 μg/mL) significantly inhibited the UVA-induced increase in the TYR, TRP1, and TRP2 protein expression [45]. A recent report marked that TSA can significantly reduce the high mRNA expression of *TYR*, *TRP1*, and *TRP2* induced by the α-MSH and the high expression of the TYR protein induced by the α-MSH [17]. Our results in this study supported previous findings that the EGCG, GCG, TFDG, and TSA reduced the amounts of enzymes in the cell model by downregulating the mRNA and protein expressions of TYR, TRP1, and TRP2 stimulated by the α-MSH (inhibiting the production of new TYR, TRP1, and TRP2 in cells), thereby reducing melanin production. The downregulating ability of catechins in the TYR family was stronger than that of dimers at both the transcription and translation levels, which accorded closely with the phenotype and the MITF expression (mRNA and protein). This fully proved that MITF is a key transcription factor regulating the *TYR* family. At the same time, the expression of the *TYR* family directly controls the TYR activity in cells.

## 4. Materials and Methods

### 4.1. Chemicals and Reagents

Pharmacological agents were EGCG (98%, Yuanye, Shanghai, China), GCG (98%, Yuanye, Shanghai, China), TFDG (99.12%), and TSA (91.4%) as test samples (generous gifts from the Tea Research Institute of Chinese Academy of Agricultural Sciences, Hangzhou, China) and kojic acid (99%, Macklin, Shanghai, China) as a positive control. 

Unless specified otherwise, all chemicals and laboratory ware used for cell culture were purchased from GIBCO (Grand Island, NY, USA), Corning (Corning, NY, USA), GENOM (Jiaxing, China), and Beyotime (Shanghai, China). α-MSH (A1025) was purchased from Apexbio (Houston, TX, USA), and L-DOPA was purchased from Macklin (Shanghai, China). EASY spin Plus cell RNA rapid extraction kit was purchased from Aidlab (Beijing, China). The reverse transcription kit (MR05201M) and fluorescence quantitative kit (MQ00401S) were purchased from Monad (Suzhou, China). Primary antibodies against TYR (TU343218), TRP1 (TD13325), TRP2 (TA5303), MITF (TU326886), p-MITF (Ser180, TA3027), and MC1R (TD4992) were purchased from Abmart (Shanghai, China). Primary antibodies against glyceraldehyde-3-phosphate dehydrogenase (GAPDH, AG019), secondary antibodies of goat anti-mouse IgG labeled with horseradish peroxidase (HRP), goat anti-rabbit IgG labeled with HRP, and other relevant reagents used for protein expression detection were purchased from Beyotime (Shanghai, China).

### 4.2. Cell Culture

B16F10 murine melanoma cells from Stem Cell Bank (Chinese Academy of Sciences, Shanghai, China) were cultured in DMEM supplemented with 10% fetal bovine serum (FBS, Gibco, Grand Island, NY, USA) in a humidified atmosphere of 5% CO_2_ at 37 °C. B16F10 cells at the exponential phase were adjusted cell density and used in the follow assay.

### 4.3. Effects of Samples on Cell Viability

A total of 100 μL of B16F10 cell suspension (1 × 10^5^ cells/mL) was seeded in flat-bottom 96-well cell culture plates. Twenty-four hours later, the culture medium was replaced with complete medium containing samples (EGCG, GCG, TFDG, or TSA). B16F10 cells were pretreated with samples for 2 h. Then they were further cultured for 48 h (cells without α-MSH stimulation) or further stimulated with α-MSH (10 µL, 1 µM) for 48 h in the presence of a sample (cells with α-MSH stimulation). After rinsing once with D-PBS, 100 µL DMEM containing 10% CCK-8 reagent was added to each well. After 1 h incubation, the absorbance at 450 nm was measured using a Synergy H1 microplate reader (BioTek Instruments, Inc., Winooski, VT, USA). The final concentration of EGCG, GCG, TFDG, TSA, and kojic acid was 100 μg/mL. All experiments were performed in triplicate.

In cells without α-MSH stimulation, a blank control group (no cells and no samples), negative control group (with cells and no samples), and sample groups (with cells and samples) were set. With the negative control group as reference (100%), cell viability of each sample group was calculated.

In cells with α-MSH stimulation, a blank control group (no cells, samples and α-MSH), CK1 (with cells, without α-MSH and samples), CK2 (with cells and α-MSH, without samples) and sample groups (with cells, α-MSH, and samples) were set. With CK2 as reference (100%), cell viability of each sample group was calculated.

### 4.4. Effects of Samples on Intracellular Melanin Content

After seeding and culturing in flat-bottom 24-well cell culture plates (0.5 mL, 1 × 10^5^ cells/mL) for 24 h, the old medium was replaced with a fresh complete medium containing the samples (100 μg/mL EGCG, GCG, TFDG, or TSA). Two hours later, 50 µL of 1 µM α-MSH was added only to the wells with cells intended for the α-MSH stimulation. The intracellular melanin content was detected after 48 h using our previous protocol [25].

### 4.5. Effects of Samples on Intracellular TYR Activity

B16F10 cells were treated with the compounds and α-MSH as in Section 4.4. The intracellular TYR activity was assayed using our previous protocol [25].

### 4.6. Effect of Samples in a Non-Cellular System on the Catalytic Activity of TYR Extracted from B16F10 Cells

B16F10 cells at the exponential phase were collected, cracked, and centrifuged. Then, the obtained lysate supernatant was used as a crude TYR solution and stored at −80 °C. The concentration of the samples was 100 μg/mL. 

The reaction system in the 96-well plate was 100 µL. A total of 20 μL of TYR was mixed with 20 μL samples and incubated at 25 °C for 10 min, then 60 μL of 8 mM L-DOPA was added to start the reaction. After reacting at 37 °C for 1 h, the absorbance at 475 nm was detected by a Synergy H1 microplate reader. A total of 20 µL of PBS (20 mM, pH 6.8) was used to replace the sample in the negative control group. A blank control group (20 µL of TYR and 80 µL of PBS) was set to exclude the interference of the crude TYR solution. A sample control group (20 µL of TYR, 20 µL of sample and 60 µL of PBS) was set to exclude the interference of the sample on the enzymatic chromogenic reaction.

### 4.7. qPCR Analysis

Using *β-actin* as internal reference, qPCR analysis was performed on *TYR*, *TRP1*, *TRP2*, *MITF*, and *MC1R* to determine the mRNA levels of these genes under different treatments. The nucleotide sequences of qPCR primers are shown in Table S3. 

B16F10 cells were pretreated with samples (100 μg/mL) for 2 h and stimulated with 100 nM α-MSH for a period of time in the presence of samples in 6-well cell culture plates (2 mL, 1 × 10^5^ cells/mL). Total RNAs extraction, reverse transcription, and qPCR analysis were carried out according to our previous protocol [25].

### 4.8. Western Blot Analysis

Western blot was performed following the method described previously with slight modifications [50,51]. Briefly, 2 mL of B16F10 cell suspension (1 × 10^5^ cells/mL) was seeded in 6-well cell culture plates. Twenty-four hours later, the culture medium was replaced with a complete medium containing the compounds (100 μg/mL). After two hours, 200 µL of 1 µM α-MSH was added into each well, and cells were further cultured for 1 h, 4 h, or 24 h. Then, cells were rinsed twice with pre-cooled D-PBS, followed by the lysis with the radioimmunoprecipitation assay (RIPA) buffer containing protease and phosphatase inhibitors on ice for 20 min. Protein lysate was collected and centrifuged at 14,000 rpm for 10 min at 4 °C, and the supernatants was collected. 

A bicinchoninic acid (BCA) protein assay kit was used to determine the total protein content after each treatment. Proteins in the supernatants were then mixed with a quarter volume of a loading buffer (5×) and heated at 100 °C for 5 min. Equal amount of protein was separated by sodium dodecyl sulfate polyacrylamide gel electrophoresis (SDS-PAGE) and transferred to polyvinylidene difluoride membranes (PVDF). After washing with a washing buffer, the membrane was incubated with a blocking buffer at room temperature for 15 min. Then the membrane was incubated with primary antibodies to TYR (1:5000), TRP1 (1:5000), TRP2 (1:5000), MITF (1:4000), p-MITF (1:4000), MC1R (1:4000), or GAPDH (1: 1000) at room temperature for 1 h, followed by washing and reacting with secondary antibodies at room temperature for 1 h. Secondary antibodies were goat anti-rabbit or goat anti-mouse IgG labeled with HRP (1:2000). Protein bands were detected by an enhanced chemiluminescence (ECL) reagent and captured using the Tanon imaging system (Tanon 5200, Tanon, Shanghai, China) after the membrane was completely washed with the washing buffer.

### 4.9. Docking Study

#### 4.9.1. Protein Pretreatment

The crystal structure of MC1R (7F4H) protein was obtained from the RCSB PDB database. The obtained MC1R crystal was subjected to a series of treatments using the Protein Preparation Wizard module of the Schrödinger software (Schrödinger Maestro 13.5), including protein preprocessing, regenerating states of native ligand, H-bond assignment optimization, protein energy minimization, and removal waters.

#### 4.9.2. Ligand Pretreatment

The 2D.sdf files of EGCG (ID: 65064), GCG (ID: 199472), TFDG (ID: 3589471), and TSA (ID: 442543), downloaded from the PubChem database, were processed by the LigPrep module of the Schrödinger software to convert to corresponding 3D chiral conformations.

#### 4.9.3. Active Site Recognition

The optimal binding site between protein and ligand was first predicted using the SiteMap module of the Schrödinger software. Then the Receptor Grid Generation module of the Schrödinger software was used to set the most appropriate Enclosing box to perfectly wrap the predicted binding site, and on this basis, the active site of MC1R protein was obtained.

#### 4.9.4. Molecular Docking

Molecular docking (XP docking with the highest precision) of the pretreated four ligand compounds with the active site of MC1R protein was performed. The lower XP Gscore indicated there was lower binding free energy and higher binding stability of the compound to the protein.

#### 4.9.5. MM-GBSA Analysis

The pretreated four ligand compounds and the active site of MC1R protein were analyzed by MM-GBSA calculation. MM-GBSA dG Bind can approximately represent the binding free energy between ligand and protein. The lower the binding free energy, the higher the binding stability between ligand and protein.

### 4.10. Statistical Analysis

The results were expressed as the mean ± standard deviation (SD) or mean ± standard error of mean (SEM). Comparisons between the two groups were performed using Student’s *t* test, and one-way analysis of variance with Duncan’s post hoc test was performed to measure the significant differences among multiple comparisons between compound effects (*p* < 0.05 (*, #, or letters), *p* < 0.01 (** or ##), and *p* < 0.001 (*** or ###) were used to consider statistical significance).

## 5. Conclusions

**Figure 7:**
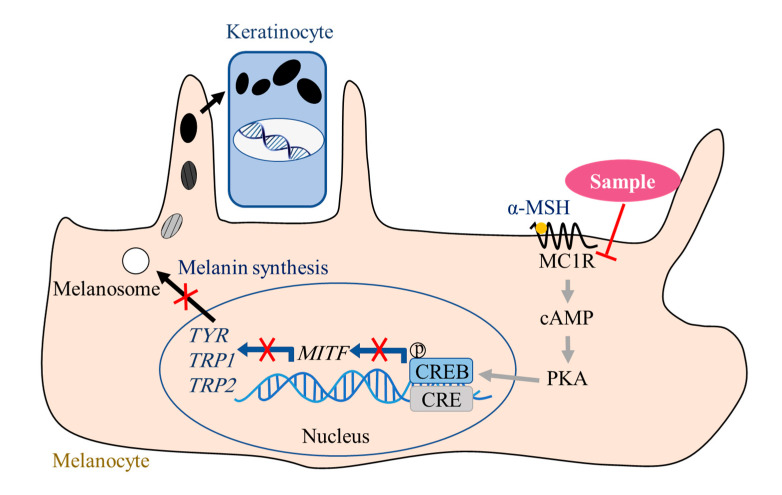
EGCG, GCG, TFDG, or TSA inhibiting melanin synthesis by regulating cyclic adenosine monophosphate-dependent signaling pathway starting at MC1R. The yellow and blue cells are melanocyte and keratinocyte, respectively. The solid orange circle stands for α-MSH. The red line and the red x represent downgrades or suppressions.

The most important finding in the present study was that, in the α-MSH-stimulated B16F10 cells, the EGCG, GCG, TFDG, or TSA could inhibit melanin formation through downregulating the expression of the MC1R protein (Figure 7), which was verified by the stable binding between these four samples and the MC1R protein in molecular docking. In addition, this study provided a new basis for the effectiveness and practicability of the α-MSH-induced cell model in research related to the anti-melanogenic activity of the samples. Then, the relationship between the anti-melanogenic activity of the samples and the inhibition of the TYR catalytic activity or the inhibition of the new enzyme synthesis related to melanogenesis were clarified. Finally, the sample activities were compared at the molecular level to verify the phenotypic results. In future research, considering the importance of MC1R, our results need to be further verified by other molecular technologies, such as mutation or overexpression of the MC1R gene. Animal experiments must also be carried out to verify the effectiveness of the EGCG, GCG, TFDG, and TSA in resisting the UV-induced tanning reactions. Through the above research, the molecular basis for the anti-melanogenic activity of the EGCG, GCG, TFDG, and TSA, as potential effective components against the UV-induced tanning reactions, was enriched. Thus, it can promote the development of tea deep processing and increase the additional value of the tea industry.

## Figures and Tables

**Table 1:** XP and MM-GBSA results.

Compound	CAS	Target	XP Gscore	MM−GBSA dG Bind (kcal/mol)
TFDG	3589471	MC1R	−10.121	−35.1
EGCG	65064		−7.844	−36.3
GCG	199472		−8.98	−27.47
TSA	442543		−9.805	−23.34

Note: Epigallocatechin gallate (EGCG), gallocatechin gallate (GCG), theaflavine-3,3’-digallate (TFDG), theasinensin A (TSA), and melanocortin 1 receptor (MC1R).

## Data Availability

Data are contained within the article and supplementary materials.

## Supplementary Materials

The supporting information can be downloaded at https://www.mdpi.com/article/10.3390/ijms241311017/s1.
