# Supplementary material for: EGCG, GCG, TFDG, or TSA Inhibiting Melanin Synthesis by Downregulating MC1R Expression
Source: Int J Mol Sci. 2023 Jul 3;24(13):11017. doi: 10.3390/ijms241311017 (PMC10341940; doi:10.3390/ijms241311017)
Supplement: Supplementary file 1 [file ijms-24-11017-s001.zip › ijms-2419535-supplementary.pdf]

## Supplementary Data

**Table S1.** Effect of EGCG, GCG, TFDG, and TSA on cell viability in B16F10 cells induced by α-MSH

| Sample | CK1 | Kojic acid | EGCG | GCG | TFDG | TSA |
|---|---|---|---|---|---|---|
| Cell viability | 96 ± 5 | 101 ± 1 | 109 ± 4 | 125 ± 4 | 115 ± 10 | 136 ± 5 |

Note: The treatment group without α-MSH and sample was referred to as control 1 (CK1), and the treatment group with α-MSH but without sample was referred to as control 2 (CK2). B16F10 cells were pretreated with complete medium, kojic acid, EGCG, GCG, TFDG, or TSA for 2 h and further stimulated with α-MSH for 48 h in the presence of complete medium, kojic acid, EGCG, GCG, TFDG, or TSA. Concentration of kojic acid, EGCG, GCG, TFDG and TSA were 100 μg/mL. Using cell viability of CK2 as a reference (100%), cell viability was greater than 90% for each treatment, implying that α-MSH stimulation did not affect cell viability and that these compounds were not toxic to α-MSH-stimulated cells at 100 μg/mL.

**Table S2.** Effects of 100 μg/mL EGCG, GCG, TFDG, and TSA on the TYR catalytic activity

| TYR catalytic activity（%） | | | | | |
|---|---|---|---|---|---|
| Control | EGCG | GCG | TFDG | TSA | Kojic acid |
| 100 | 96 ± 1 | 96 ± 1 | 98 ± 2 | 99 ± 2 | 29 ± 1 |

Note: The treatment group without samples was used as control (TYR catalytic activity was 100%). In the non-cellular system, the effect of samples on the activity of L-DOPA oxidation by TYR was observed.

**Table S3.** Nucleotide sequences of qPCR primers.

| Gene | ID | Primer sequence（5' to 3'） | Tm | GC% | Accession Number |
|---|---|---|---|---|---|
| *β-actin*-F | 11461 | TTGACATCCGTAAAGACCTCTATGCC | 61.8 | 46.2 | NM_007393.5 |
| *β-actin*-R | 11461 | ACCGATCCACACAGAGTACTTGC | 61.7 | 52.2 | |
| *TYR*-F | 22173 | CATGGTTCCTTTCATACCGCTCT | 59.3 | 47.8 | NM_011661.5 |
| *TYR*-R | 22173 | TCTGTAAAAGCCTGGATCTGACT | 59.6 | 43.5 | |
| *TRP1*-F | 22178 | TGGAAAACGCACCTATTGGACA | 60.4 | 45.5 | NM_031202.3 |
| *TRP1*-R | 22178 | TAACAACGCAGCCACTACAGC | 60.8 | 52.4 | |
| *TRP2*-F | 13190 | ATAAAGCAGACGGAACACTGGACT | 63.6 | 45.8 | NM_010024.3 |
| *TRP2*-R | 13190 | ACCACAAACACAGGGTCGTT | 62.5 | 50 | |
| *MITF*-F | 17342 | CGCATGGACTTTCCCTTATCCC | 60.7 | 54.5 | NM_008601.3 |
| *MITF*-R | 17342 | CCTGGTGCTGTACAAGTTCCT | 60.7 | 52.4 | |
| *MC1R*-F | 17199 | CTGTATGCCCACATGTTCACGAG | 62.3 | 52.2 | NM_008559.2 |
| *MC1R*-R | 17199 | CCCAGCACAGGAAGAAAATCCCC | 62.8 | 56.5 | |

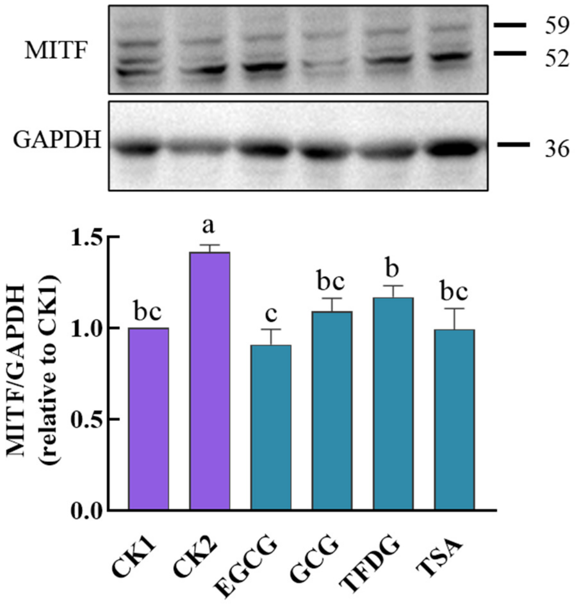


**Figure S1.** Effect of EGCG, GCG, TFDG, or TSA on expression of MITF protein after stimulated by α-MSH for 1 h. The treatment group without α-MSH and sample was referred to as CK1, and the treatment group with α-MSH but without sample was referred to as CK2. B16-F10 cells were pretreated with EGCG, GCG, TFDG or TSA for 2 h and stimulated with α-MSH for 1 h in the presence of EGCG, GCG, TFDG or TSA. Protein extracts were prepared by lysing cells with RIPA lysate containing protease inhibitors and phosphatase inhibitors. Total proteins were subjected to western blot analysis of MITF with GAPDH as an internal control. Data are mean ± SEM. [a,b,c] Different letters above the column indicate significant differences ($p < 0.05$).

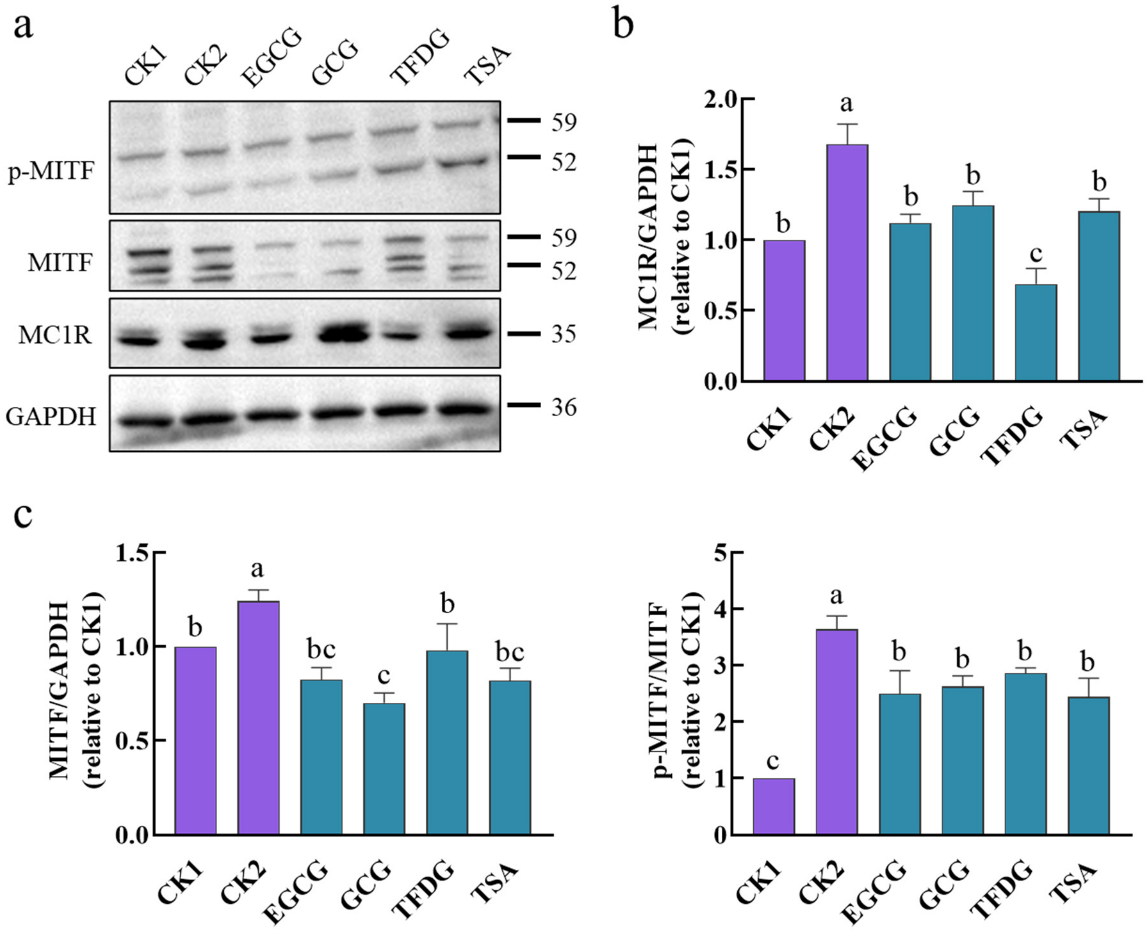


**Figure S2.** Effect of EGCG, GCG, TFDG, or TSA on expression of MITF, p-MITF and MC1R protein after stimulated by α-MSH for 4 h. The treatment group without α-MSH and sample was referred to as CK1, and the treatment group with α-MSH but without sample was referred to as CK2. B16-F10 cells were pretreated with EGCG, GCG, TFDG or TSA for 2 h and stimulated with α-MSH for 4 h in the presence of EGCG, GCG, TFDG or TSA. Protein extracts were prepared by lysing cells with RIPA lysate containing protease inhibitors and phosphatase inhibitors. Total proteins were subjected to western blot analysis of MC1R (a, b), MITF (a, c) and p-MITF (a, c) with GAPDH as an internal control. Data are mean ± SEM. [a,b,c] Different letters above the column indicate significant differences ($p < 0.05$).

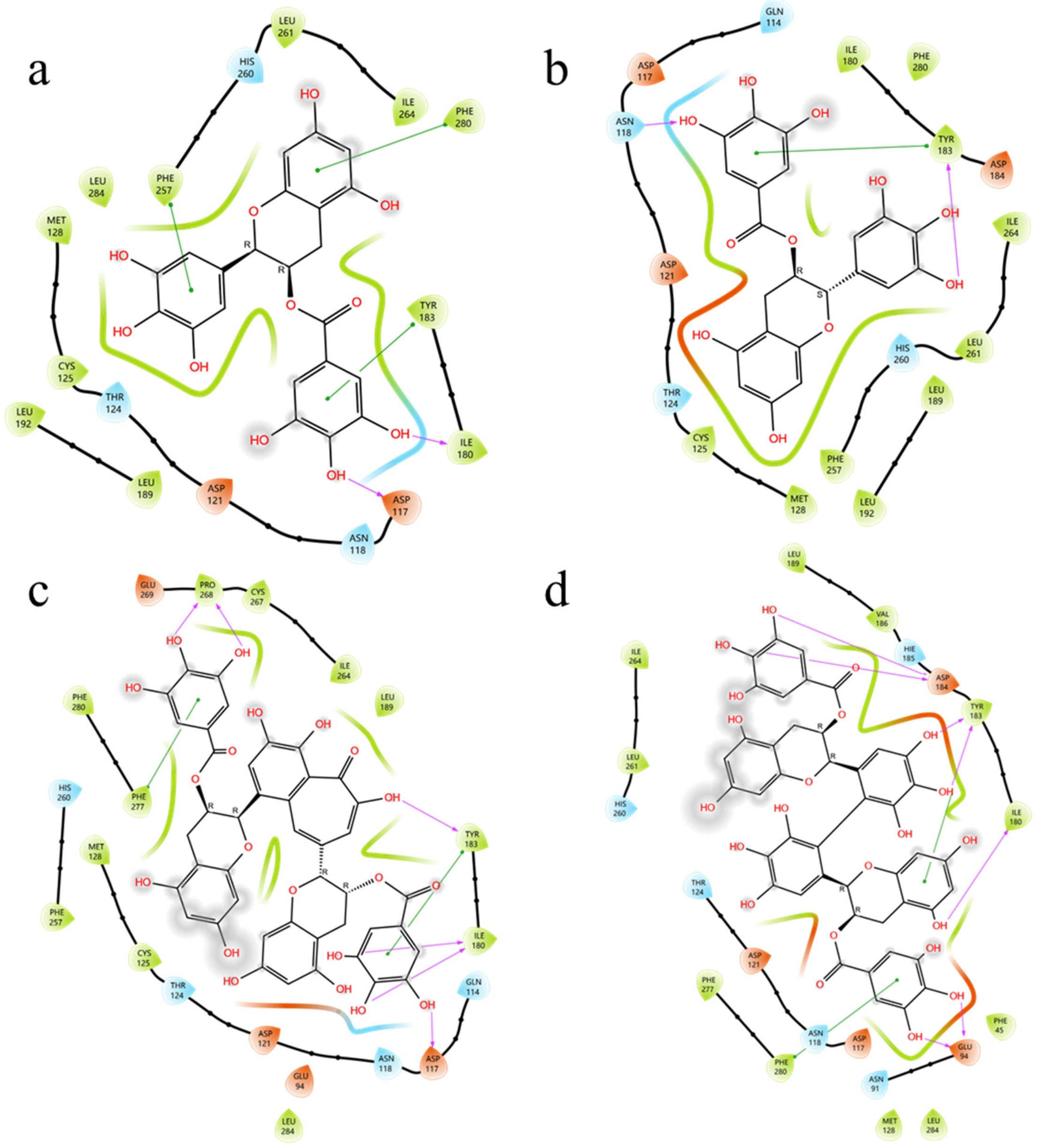


**Figure S3.** 2D images of the four compounds docking with MC1R protein. (a) EGCG; (b) GCG; (c) TFDG; (d) TSA.
